# RP-HPLC for simultaneous determination of water-soluble vitamins in leafy vegetables using ultrasonicated acid hydrolysis

**DOI:** 10.1039/d5ra04911c

**Published:** 2025-10-14

**Authors:** Tikeshwari, Kamlesh Shrivas, Khushali Tandey, Ankita Tejwani, Arun Kumar Patel, Anuradha Sharma

**Affiliations:** a School of Studies in Chemistry, Pt. Ravishankar Shukla University Raipur-492010 CG India kshrivas@gmail.com; b Chandrapal Dadsena Govt. College Pithora Mahasamund CG 493551 India; c School of Studies in Electronics, Pt. Ravishankar Shukla University Raipur-492010 CG India; d Department of Zoology, Govt. Nagarjuna P.G. College of Science Raipur-492010 CG India

## Abstract

This study reports the development and application of a rapid and efficient reverse-phase high-performance liquid chromatography (RP-HPLC) method for simultaneous separation and identification of water-soluble vitamins (WSVs) such as B_1_, B_2_, B_3_, B_6_, and B_9_ in green leafy vegetables (GLVs) from Chhattisgarh, India. A sample preparation method based on acid hydrolysis assisted by ultrasonication was optimized using 0.1 M HCl. Separation and detection of the vitamins were carried out using a C-18 column with a gradient elution of orthophosphoric acid (OPA) and methanol (MeOH), with UV detection at 270 nm. The method demonstrated excellent linearity (*r*^2^ > 0.993), low limits of detection (LODs) ranging from 0.06 to 0.15 μg mL^−1^, and high recovery rates (91.5–98.0%), confirming its sensitivity and accuracy. The analysis of 17 GLVs from the Chhattisgarh region demonstrated considerable variation in WSV content per 100 g. The concentration of WSV B_1_ ranged from 0.12 to 0.85 mg, B_2_ from 0.08 to 0.40 mg, B_3_ from non-detectable (ND) to 2.64 mg, B_6_ from 0.07 to 0.64 mg, and B_9_ from ND to 0.08 mg. Furthermore, estimated daily intake (EDI) values were calculated to assess the contribution of these GLVs toward the recommended dietary intake for both men and women. The findings suggest that regular consumption of these vegetables can significantly enhance nutritional adequacy. This method provides a robust analytical tool for the simultaneous determination of five essential vitamins in food matrices.

## Abstract 2

This study presents a rapid and efficient RP-HPLC method for simultaneous separation and identification of water-soluble vitamins (B_1_, B_2_, B_3_, B6_,_ and B_9_) in green leafy vegetables from Chhattisgarh, India.

## Graphical abstract

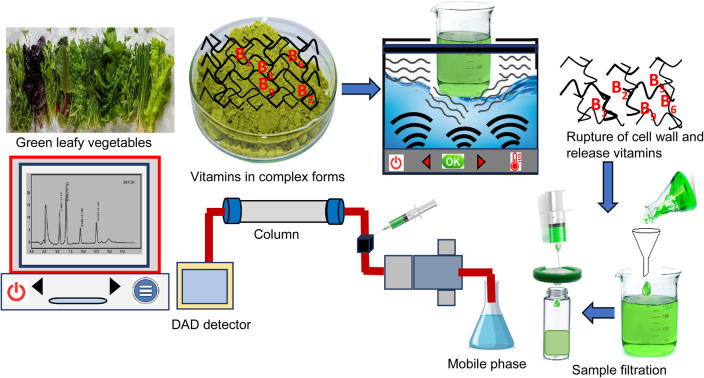

## Introduction

Vitamins are essential organic compounds required in small amounts by the human body to maintain normal physiological functions and overall health. They play a crucial role in numerous biological processes and help to prevent disorders associated with nutrient deficiencies. Vitamins are broadly classified into two main categories, such as fat-soluble vitamins (A, D, E, and K) and water-soluble vitamins, which include the B-complex group and vitamin C. Vitamins like B_1_ (thiamine), B_2_ (riboflavin), B_3_ (niacin), B_6_ (pyridoxine), and B_9_ (folic acid) are essential for maintaining proper health, and are found in foods like whole grains, dairy products, eggs, meat, fish, potatoes, bananas, leafy green vegetables, legumes, *etc.*^1,2^ Insufficient intake of these vitamins may result in several health disorders, such as beriberi, oral ulcers, visual impairments, pellagra, anaemia, dermatological issues, and congenital abnormalities like spina bifida. Therefore, it is essential to measure the levels of these vitamins in food samples to ensure adequate dietary intake. The separation and identification of vitamins in food materials are vital for understanding nutritional intake and preventing deficiencies.^3,4^

In GLVs, the concentration of vitamins is typically low, and the presence of other chemically similar compounds in the samples renders the challenging for analysis of WSVs. Analytical methods for quantifying different WSVs vary from conventional approaches such as spectrophotometry to more advanced techniques such as HPLC,^5,6^ LC-mass spectrometry (MS),^7,8^ immuno-based assays,^9^ and electrochemical methods.^10,11^ Immuno-based assays provide specificity, but may lack broad applicability to all vitamins. Electrochemical methods offer cost-effective, rapid detection but may suffer from interference in complex samples. Spectrophotometry is simple and widely available, though less sensitive and selective than chromatographic techniques. Each method has its advantages, but choosing the right one depends on the matrix, accuracy requirements, and available resources, making them valuable for nutritional analysis in GLVs. HPLC and LC-MS offer high sensitivity and accuracy, allowing for precise quantification of vitamins even in complex matrices, though they require expensive equipment and expertise.^5–8^

In HPLC analysis of food samples, the preparation of samples is a key step that often presents challenges, especially when targeting WSVs. Various techniques have been developed to address this, including enzymatic hydrolysis,^12,13^ ultrasonication,^5^ solid-phase extraction (SPE),^14^ soxhlet extraction,^15^ and acid hydrolysis, *etc.*^16,17^ These methods are used to isolate the specific WSVs in food and vegetable matrices prior to instrumental analysis. The enzymatic hydrolysis is frequently used in sample preparation, due to the enzyme's catalytic ability to break bonds between vitamins and other biomolecules. This process helps in controlled release of vitamins from complex sample matrices, simplifying their analysis.^12,13^ Arella *et al.* introduced an enzymatic method for determining vitamins B_1_ and B_2_ through HPLC analysis. In this method, powdered food samples were treated with 0.1 M HCl and heated in a water bath at 100 °C for 30 min. Following this, the solution was adjusted to a pH of 4.5 using sodium acetate (CH_3_COONa). The sample was then subjected to enzymatic hydrolysis by incubating it with p-amylase and taka-diastase at 37 °C for 18 h. After incubation, the solution was filtered using a syringe filter, and subjected to HPLC analysis. Although this method yielded reliable results, it required several procedural steps, rendering it labour-intensive and time-consuming process.^12^ Other enzymatic methods for analysing vitamins B_2_ and B_6_ in Amaranthaceous crops involved the similar steps but also posed challenges, particularly with plant-based samples.^13^ Ultrasonication is a widely used method for extraction of WSVs before HPLC analysis. It utilizes sound waves to create mechanical vibrations in a liquid solution, breaking cell walls and releasing WSVs into the solution. Patle *et al.* employed ultrasonication as an effective technique to extract WSVs from leafy vegetables. After sonication, the samples underwent centrifugation and filtration, followed by solvent removal using a rotary evaporator. The dried residue was dissolved in methanol for HPLC analysis. Although the drying step may risk the degradation of heat-sensitive vitamins, ultrasonication proved to be a robust method for extracting vitamins from plant matrices. SPE is also commonly used for sample preparation and can be highly effective for analysing WSVs.^5^ SPE aids in concentrating, purifying, and isolating vitamins from complex matrices like food, pharmaceutical, and clinical samples. Płonka *et al.* utilized SPE to extract B-complex vitamins from fruits and fruit-vegetable juices. In a typical method, 100 mL of juice was treated with a mixture of zinc acetate–acetic acid and potassium ferricyanide. The mixture was then centrifuged, and the resulting supernatant was filtered through a 0.45 μm membrane filter before being subjected to analysis. This process removed the interfering substances and resulted in cleaner extracts, improving detection limits. However, due to the high polarity of WSVs, their interaction with the SPE sorbent might be inefficient, potentially reducing recovery rates. Further soxhlet extraction (SE) was used to extract WSVs from food materials. In this process, powdered samples undergo repeated solvent extraction cycles.^14^ Khalkho *et al.* reported a SE method separation of B_1_ from vegetables and detected using silver nanoparticles (AgNPs) as a sensing probe. In SE, 10 g of the sample were subjected to reflux with 40 mL of an organic solvent for 4 h to extract B_1_. After centrifugation and filtration, the purified extract was analysed for B_1_ from food samples. Despite its effectiveness, SE was a lengthy process that needed a large volume of organic solvents and has the potential to degrade heat-sensitive vitamins.^15^ Further, acid hydrolysis is used to extract vitamins (B_1_, B_2_, B_3_, and B_6_) from food samples prior to LC-MS analysis. In this method, a 1 g sample was mixed with 25 mL of 0.1 N HCl and heated to a temperature range of 121–123 °C, with the pH adjusted using 2.0 M CH_3_COONa. This approach enabled the simultaneous analysis of multiple vitamins but carries the risk of degrading WSVs at high temperatures.^16,17^

As far as we know, we have used acid (HCl) hydrolysis combined with ultrasonication for extraction of B_1_, B_2_, B_3_, B_6_ and B_9_ in GLVs from Chhattisgarh region. In this work, acid hydrolysis method assisted with ultrasonication was employed for digestion of GLVs. The digested sample was injected into RP-HPLC for determination of vitamins. The parameters such as HCl, H_2_SO_4_, H_3_PO_4_, MeOH and enzyme, and their different combination composition that affected digestion and separation of WSVs in vegetable sample in HPLC analysis were investigated. In addition, flow rate, concentration of H_3_PO_4,_ and effect of wavelength were optimised for better separation and detection of WSVs in RP-HPLC. The method was successfully applied to analyse WSVs in GLVs from the Chhattisgarh region.

## Materials and methods

### Reagent and solution preparations

Vitamins (B_1_, B_2_, B_3_, B_6_, B_9_) and taka-diastase were obtained from Sigma-Aldrich (MD, USA). Orthophosphoric acid (OPA), hydrochloric acid (HCl), and sulfuric acid (H_2_SO_4_) were procured from Merck (Mumbai, India). HPLC-grade acetonitrile (ACN), methanol (MeOH), and water were obtained from J. T. Baker (Avantor, India). A stock standard solution (100 μg mL^−1^) of all the vitamins was prepared in HPLC-grade water.

### Plant sample collection

In the present work, different GLVs such as *Hibiscus sabdariffa* L. (Amari Bhaji), *Cordia dichotoma* (Bohar Bhaji), *Amaranthus viridis* L. (Chaulai Bhaji), *Marsilea vestita* (Chunchunia Bhaji), *Cassia tora* (L.) *Roxb.* (Charota Bhaji), *Chorchorus olitorius* L. (Chech Bhaji), *Ipomea aquatica* (Karmata Bhaji), *Carthamus tinctorius* L. (Kusum Bhaji), *Ipomea batatas* L. *Lam.* (Kanda Bhaji), *Amaranthus tricolor* L. (Lal Bhaji), *Lathyrus sativus* L. (Lakdi Bhaji), *T. foenumgraecum* L. (Methi Bhaji), *Spinacia oleracea* L. (Palak Bhaji), *Allium cepa* L. (Pyaj Bhaji), *Basella rubra* L. (Poi Bhaji), *Hibiscus sabdariffa* L. (Patwa Bhaji), *Chenopodium album* L. (Bathua Bhaji) were collected from different region of Chhattisgarh in polythene bag. The plant samples were cut into little pieces, cleaned with tap water, and then dried at room temperature. GLVs of Chhattisgarh showing local name, botanical name, family, ecological occurrence, season of availability, uses are listed in Table S1 (SI). The dried sample was grinding machine into fine powder and kept in an airtight container.^18^

### Sample preparation

Two grams of fine powder sample were taken in separate beakers, each containing 10 mL of HCl (0.1 M), H_2_SO_4_ (0.1 M), H_3_PO_4_ (1%), HCl + taka-diastase, HCl + taka-diastase, H_3_PO_4_ + taka-diastase, or H_2_SO_4_ + taka-diastase. The samples were subjected to ultrasonic treatment in an ultrasonic bath (PCI Analytics) operating at 44 kHz for 30 min at room temperature. After sonication, the sample solutions were centrifuged and then filtered through Whatman filter paper (no. 42). The filtrates were further purified using a syringe filter (0.2 μm). The extracts were then injected into HPLC for determination of WSVs.

### RP-HPLC-DAD conditions

An HPLC system (Ultimate 3000, Thermo Fisher) equipped with a diode array detector (DAD) was used for measurement of WSVs. An aliquot of the vitamin standard/sample extract (20 μL) was injected onto a C-18 column (Hypersil GOLD™, 100 × 4.6 mm × 5 μm; Thermo Fisher Scientific, Madison, USA) for vitamin quantification. The separation of WSVs were carried out using a gradient elution system at room temperature, consisting of solvent A (0.1% OPA) and solvent B (methanol, MeOH). The flow rate was set to 1.0 mL min^−1^. The gradient program was as follows: 98% solvent A from 0.1 to 1.1 min; 96% A from 1.1 to 2.4 min; 95% A from 2.4 to 5.6 min; 90% A from 5.6 to 6.2 min; 60% A from 6.2 to 14 min; 40% A from 14 to 16.4 min; and re-equilibration with 100% A from 16.4 to 20 min. The total run time was 20 min (Table S2). The detection of all WSVs was carried out at a wavelength of 270 nm.

### Analytical evaluation

The linear least square equation (*y* = *mx* + *c*) from the standard calibration curve was used to determine WSVs in GLV samples. The correlation coefficient (*r*^2^) indicated the method's linearity, with values close to 1 signifying excellent linearity for vitamin measurement. The method reproducibility was assessed by intra-day precision. The intra-day precision was evaluated by analysing a 10 μg mL^−1^ vitamin solution six times within the same day, while inter-day precision involved analysing the same concentration over six consecutive days. Precision was expressed as standard deviation (SD) and relative standard deviation (RSD%), as described by Patle *et al.*^5^ Accuracy was determined by calculating percentage recovery after spiking standard WSV solutions at 1 and 5 μg mL^−1^ into blank or real samples (Table S3), following the work of Patle *et al.* LOD and LOQ were calculated based on the minimum detectable quantity of vitamin at three and ten times the standard deviation, respectively, divided by the slope from the linear equation.

**Scheme 1:**
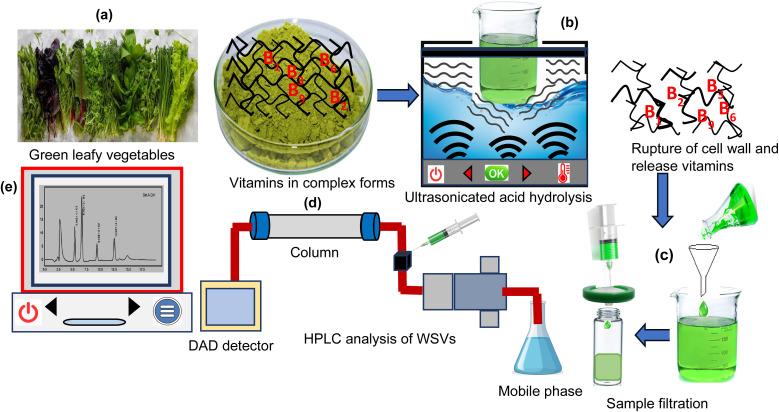
(a) Sample collection of green leafy vegetables, (b) ultrasonication, rupture of cell walls and release of vitamins, (c) filtration, (d) HPLC analysis of vitamins B_1_, B_2_, B_3_, B_6_, and B_9_, and (e) spectrum of a WSVs.

Scheme 1(a–e) outlines the stepwise procedure for the analysis of WSVs in GLVs. The process begins with the collection of fresh GLV samples, followed by ultrasonication, which facilitated the rupture of plant cell walls and efficient release of vitamins. The resulting extract was then subjected to filtration to remove particulates. Finally, the filtrate was analysed using RP-HPLC for simultaneous quantification of essential vitamins B_1_, B_2_, B_3_, B_6_, and B_9_.

Estimated daily intake (EDI). Vitamins play crucial roles in metabolism, immunity, and overall health. The monitoring of EDI and maintaining optimal vitamin intake is essential for long-term health and disease prevention. The EDI of a vitamin is calculated using the following formula:
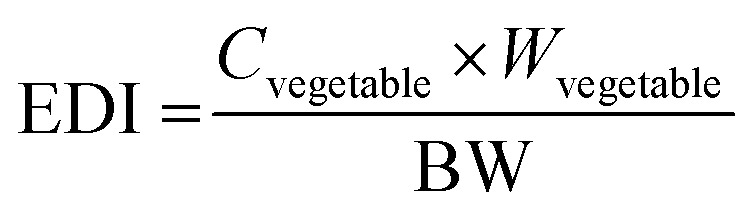
Here, *C*_vegetable_ is the concentration of vitamins in vegetables (mg kg^−1^), *W*_vegetable_ is the daily intake of vegetables (0.1 kg), and BW is the average body weight of men (65 kg) and women (55 kg) according Indian Council of Medical Research (ICMR).^19^

## Results and discussion

The analysis of WSVs using RP-HPLC-DAD is challenging due to their differing polarity, stability, and solubility, and their occurrence in complex GLVs matrices. In the present work, C-18 column was selected for its non-polar stationary phase, suitable for polar vitamin separation. The use of low ionic strength and acidic mobile phases enhanced selectivity. The gradient elution improved retention and separation, enabling accurate quantification of WSVs in complex samples.^5,20,21^

### Optimization of instrumental parameters in HPLC for analysis of WSVs

Optimization of instrumental parameters, such as wavelength, flow rate, and retention time, were essential in HPLC analysis of WSVs. The proper selection of these parameters ensured the peak resolution, quantification, improving sensitivity, reproducibility, and overall method efficiency.^5,21^ Further, wavelength selection (210, 270, and 390 nm) in DAD detector played an important role in optimizing the detection of vitamins, as it significantly influenced sensitivity and specificity, as illustrated in Fig. S1(a)–(c). At 210 nm, although all five vitamins were detectable, the resolution was poor, and peak tailing was observed, particularly at higher retention times. In contrast, 270 nm provided well-resolved, distinct peaks with a stable baseline for all vitamins, ensuring both clarity and accuracy. At 390 nm, only B_2_ and B_9_ could be detected, while the other vitamins showed no response. Overall, 270 nm was identified as the optimal wavelength, better separation and identification of all WSVs.

The flow rate had a significant impact on the detection of vitamins using RP-HPLC-DAD, as supported by findings in Fig. S2(a) to S2(c).^5,21^ A flow rate of 1.0 mL min^−1^ provided the optimal balance between resolution and efficiency of WSVs, yielding sharp peaks with high absorbance and consistent retention times. At a lower flow rate of 0.5 mL min^−1^, poor resolution and overlapping peaks were observed, likely due to excessive diffusion and prolonged interaction time, which impaired separation and identification of the vitamins. In contrast, raising the flow rate to 1.5 mL min^−1^ noticeably shortened the retention time, thereby reducing the interaction between stationary phase and analytes. This caused peak broadening and a noticeable drop in resolution and sensitivity. Overall, the study demonstrated that 1.0 mL min^−1^ was the optimal flow rate for detection of WSVs.

**Fig. 1:**
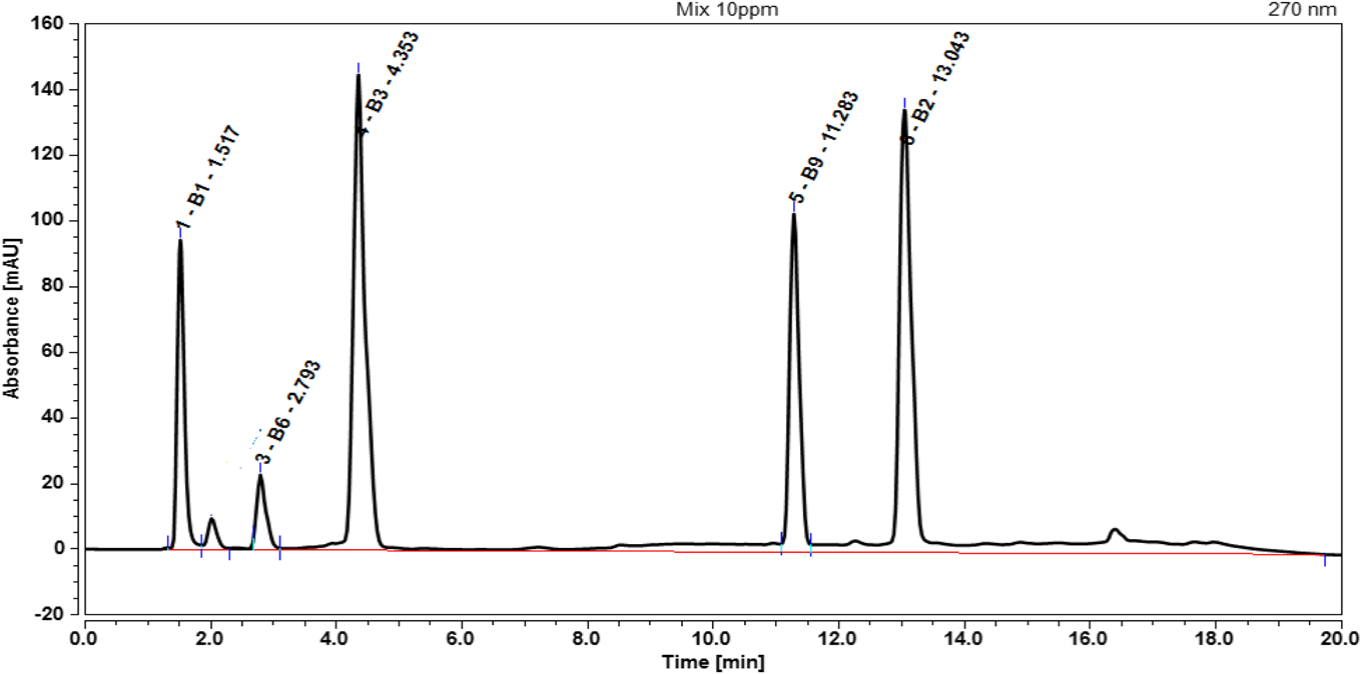
Chromatographic peaks of standard B_1_, B_2_, B_3_, B_6_, and B_9_ (5 μg mL^−1^) vitamins with their retention time of 1.51, 13.04, 4.35, 2.79 and 11.28 min, respectively, at flow rate of 1.0 mL min^−1^ and detection at 270 nm.

Furthermore, retention times play a key role in detection and resolution of WSVs. The study demonstrated that each vitamin exhibited distinct retention times: B_1_ at 1.51 min, B_6_ at 2.79 min, B_3_ at 4.35 min, B_9_ at 11.28 min, and B_2_ at 13.04 min, shown in Fig. 1. These retention times allowed clear separation of the vitamins, minimizing overlap and ensuring accurate detection. The range of retention times provided sufficient interaction between the stationary phase and analytes, achieving good resolution without excessive analysis time. This balance indicated that the given retention times were optimal for both efficient separation and quantification of vitamins in the HPLC analysis.

### Optimization of experimental conditions for analysis of WSVs

Optimization of experimental conditions for WSV analysis involved fine-tuning the mobile phase composition such as solvent A (OPA) and solvent B (MeOH). The OPA-to-MeOH ratio significantly affected the separation efficiency and resolution of WSVs, while OPA concentration was critical for maintaining consistency and ensuring proper separation of the analytes.^22^ The gradient program for the elution of WSVs is given in Table S2, facilitated the separation of vitamins. The gradient flow started with 98% OPA (A) and 2% MeOH (B) at 0.0 min, gradually reducing the OPA percentage while increasing the MeOH content. This approach allowed for efficient elution of the vitamins over a 20 min run time. Specifically, from 0–1.1 min, the system was 98% OPA, helping retain the more polar WSVs. The proportion of methanol was gradually increased from 2% to 60% between 1.1 and 15 min, promoting the elution of less polar compounds. Finally, the system returned to 100% OPA from 20 min onwards to complete the run and flush the column. This gradient elution program was effective for achieving better separation and determination of the vitamins, optimizing both the sensitivity and resolution.

The effect of OPA showed a significant impact on the vitamin chromatograph. Three different OPA concentrations, such as 0.05%, 0.1%, and 0.2%, were selected for analysis of WSVs, shown in Fig. S3(a) to S3(c). At 0.05% and 0.2% OPA, various peaks of coeluted components appeared alongside the vitamins, complicating the analysis. However, only 0.1% OPA provided sharp and distinct absorption peak, which facilitated better separation of vitamins. As a result, 0.1% OPA was selected as the optimal mobile phase concentration, offering enhanced sensitivity and peak resolution in HPLC analysis of vitamins.

### Optimization of sample digestion for analysis of WSVs

**Fig. 2:**
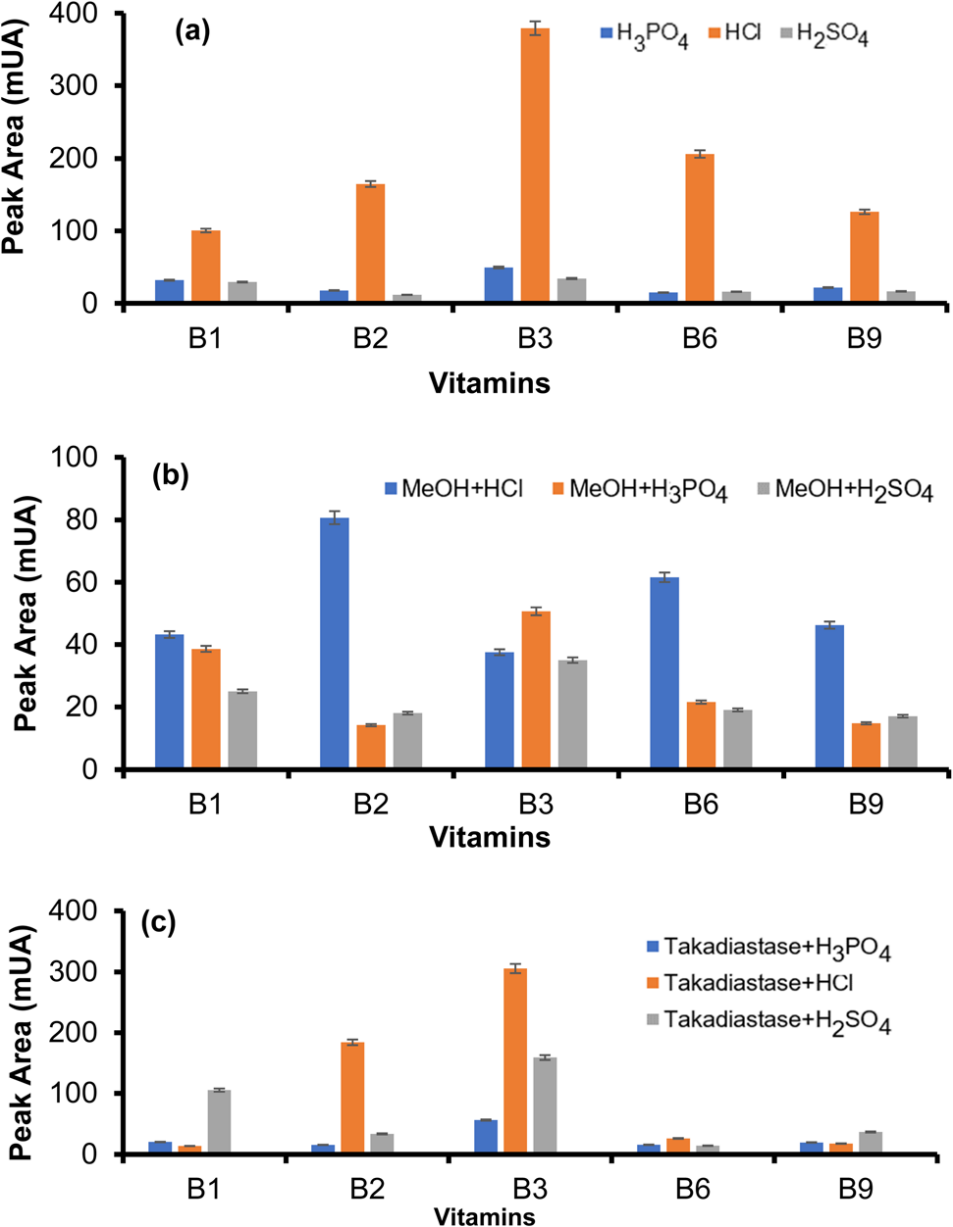
(a) Effect of a different acids (HCl, H_2_SO_4_, H_3_PO_4_), (b) effect of MeOH with acids (HCl, H_2_SO_4_, H_3_PO_4_), and (c) effect of enzyme with acids (HCl, H_2_SO_4_, H_3_PO_4_) for digestion of WSVs and analysis in HPLC.

Herein, different acids (HCl, H_2_SO_4_, H_3_PO_4_), varying incubation times (10–60 min), and HCl concentrations (0.02–0.2 M) for effective separation of WSVs. The conditions were adjusted to enhance hydrolysis efficiency and vitamin stability.^16,17^ The type of acid used in sample preparation played a critical role in the efficiency of extracting WSVs from GLVs, especially when coupled with ultrasonication and HPLC analysis. Among the acids tested (0.1 M HCl, H_2_SO_4_, H_3_PO_4_), HCl demonstrated superior results, yielding higher signal intensity for all analyzed WSVs, illustrated in Fig. 2(a). HCl demonstrated the superior performance in digestion of GLVs because its strong acidity effectively disrupted the plant cell walls, promoted solubilization, and released bound B-vitamins. In addition, the combination of ultrasonication and HCl enhanced extraction efficiency, yielding higher signal intensity, better recovery, and minimal matrix interference compared to other acids. Further, a solution mixture of MeOH with different acids was tested for the extraction of WSVs to evaluate the synergistic effect of MeOH and HCl.^23^ As shown in Fig. 2(b), the combination of MeOH with HCl yielded lower signal intensity than HCl alone, indicating reduced extraction efficiency in the presence of methanol. Consequently, methanol with HCl was not considered from further use in the digestion protocol for WSVs. In Fig. 2(c), the extraction of vitamins from GLVs was investigated using a combination of the enzyme Taka-diastase with various acids (such as HCl, H_3_PO_4_ and H_2_SO_4_), coupled with ultrasonication.^24,25^ Taka-diastase, combined with these acids, enhanced WSV extraction by breaking down complex matrices and releasing the vitamins. In the present study, the combination of taka-diastase with various acids resulted in lower extraction efficiency compared to the use of HCl alone. Therefore, HCl was selected as the preferred for digestion of GLVs to achieve better separation of WSVs prior to HPLC analysis.

### Analytical evaluation for determination of WSVs

The analytical assessment for quantifying WSVs, *via* HPLC was conducted by evaluating parameters like linearity, LOD, LOQ and precision. The results are presented in Table 1. Linearity was evaluated by spiking various concentrations of WSVs into sample and measuring the resulting responses.^5^ A five-point calibration curve was constructed for each WSVs with concentration ranges of 0.25–20 μg mL^−1^ for B_1_, 0.2–20 μg mL^−1^ for B_2_ and B_3_, 0.5–20 μg mL^−1^ for B_6_, and 0.4–20 μg mL^−1^ for B_9_, as shown in Fig. S4. The correlation coefficient (*r*^2^) of the method for measurement of these vitamins were in the range between 0.996 and 0.999, reflecting excellent linearity across all target analytes. The method also demonstrated remarkable sensitivity, with LOD between 0.06–0.15 μg mL^−1^ and LOQ between 0.20–0.50 μg mL^−1^. These values confirmed that the method's ability to accurately quantify trace levels of vitamins in complex matrices.

**Table 1:** Summary for analytical evaluation for determination of WSVs

Vitamins	Linearity range, μg mL^−1^	*r* ^2^	LOD, μg mL^−1^	LOQ, μg mL^−1^	RSD ± %	Recovery, %
B_1_	0.25–20	0.998	0.08	0.25	1.1	94.0–96.0
B_2_	0.2–20	0.996	0.06	0.20	2.2	96.5–98.0
B_3_	0.2–20	0.999	0.06	0.20	1.5	91.0–94.0
B_6_	0.5–20	0.997	0.15	0.50	2.5	93.5–98.0
B_9_	0.4–20	0.998	0.10	0.30	1.3	92.0–93.0

The RSD values for intra-day and inter day precision for vitamin determination ranged from 1.1 to 2.5% and 2.7 to 4.6% respectively. The low RSD values (<5%) indicated the high precision and reliability, confirming the method's suitability for WSV quantification in complex matrices. Overall, the combination of strong linearity, low detection limits, and high precision demonstrated the robustness of this HPLC method for accurately quantifying WSVs in complex matrices.

The determination of recovery percentage is essential for evaluating the accuracy of the developed HPLC method for analyzing WSVs in GLVs. Recovery was calculated as the ratio of spiked concentrations (0.5 and 2 μg mL^−1^) to the amount found after spiking in pretreated vegetable samples. As shown in Table S3, recoveries ranged from 91.5% to 98.0%, demonstrating high method reliability. These values confirmed the minimal matrix interference and efficient extraction, ensuring accurate vitamin quantification. Overall, better recovery percentages and accuracy, making it suitable for WSVs analysis in complex vegetable matrices for nutritional and quality assessments.

### Comparative evaluation of the developed HPLC method for WSVs determination with reported methods

A comparison of current HPLC method with previously established methods for determining WSVs demonstrated its better efficiency, and simplified sample preparation (Table 2). Many existing methods utilized the complex mobile phases, such as trifluoroacetic acid (TFA) and phosphate and acetate buffers, requiring extensive pH adjustments and buffer preparation.^21,26–28^ In contrast, the present method employed a simpler mobile phase of OPA and MeOH, minimizing reagent complexity while maintaining effective separation. Further, a key advantage of this method is its rapid and efficient sample preparation. While previous methods require long enzymatic hydrolysis (2–16 h)^8,28,29^ or incubation in phosphate/acetate buffers for 30 min to 24 h.^21,26,30–32^ The present method utilized the ultrasonication with 0.1 M HCl for just 30 min, without use of buffer, and reducing digestion time. Furthermore, the present method is simple for determination of WSVs in GLVs compare to previous studies which focused on energy drinks, supplements, or cereals, broadening its applicability in food analysis.

**Table 2:** Comparison for determination of WSVs in several samples using HPLC with other reported methods

WSVs	Mobile phase/pH/run time	Linearity range, μg mL^−1^	LOD, μg mL^−1^	Sample digestion method	Sample	Ref.
B_1_	A: Phosphate buffer	0.01–10	0.03	Sample in water + ultrasonication	Green leafy vegetables	5
B_3_	B: ACN pH: 3.0	0.01–10	0.03			
B_6_	Elution: Gradient, 7 min	0.01–10	0.03			
B_1_	A: Water	0.05–9	0.003	Sample in sodium acetate (pH 4.5) + glyoxylic acid, l-glutathione, EDTA, Fe(ii) sulfate, and enzyme mixture, then incubated at 37 °C for 14 h	Wheat flour	8
B_2_	B: MEOH +0.1% formic acid		0.001			
B_6_	Elution: Gradient		0.002			
	LC-MS					
B_1_	A: NH_4_COONa buffer	0.005–10	0.001	Sample hydrolysed in 0.1 M HCl + water bath for 30 min at 121–123 °C + adjusted with 2 M ammonium acetate	Pasta	16
B_2_	B: MEOH, (pH 3.75)	0.005–12.5	0.005			
B_3_	Gradient elution, LC-MS, 12 min	0.01–25	0.005			
B_6_	LC-MC	0.05–25	0.002			
B_9_		0.01–50	0.005			
B_1_	A: ACN	20–100	0.03	Sample in 0.1 M NaOH + phosphate buffer (1 M) + 24 h	Wild edible fruits	21
B_2_	B: TFA pH: 5.5	20–100	0.2			
B_3_		20–100	0.3			
B_6_	Elution: Gradient, 35 min	20–100	1.1			
B_9_		20–100	0.3			
B_2_	A: TFA	0.01–100	0.3	Sample in water + NaOH + phosphate buffer	Honey	26
B_3_	B: ACN pH: 5.5	0.05–500	0.3			
B_9_	Elution: Gradient, 20 min	0.05–100	0.6			
			0.2			
B_1_	A: MeOH	0–50	0.03	Sample in water + ultrasonication	Energy drinks and vitamin supplement	27
B_2_	B: NaH_2_PO_4_ + hexane sulfonic acid pH: 3.0	0–50	0.06			
B_3_		0–50	0.06			
B_6_	Elution: Gradient, 30 min	0–50	0.02			
B_9_		0–10	0.02			
B_1_	A: TFA	0.05–1.0	0.010	Sample digested in 0.1 M HCl, ultrasonic and water bath + pH adjustment + taka-diastase addition, TCA treatment, followed by buffer-2 h	Baby foods	28
B_2_	B: ACN pH: 2.6	0.02–1.0	0.003			
B_3_		0.20–1.5	0.04			
B_6_	Elution: Gradient	0.10–1.5	0.02			
B_9_		0.02–1.0	0.005			
B_1_	A: H2O + ACN (95 : 5)	0.08–30	0.08	Enzymatic hydrolysis with 0.1 HCl, 16 h, 0.1 M HCl + incubated at 100 °C for 30 min. Adjustment pH 4.5 for 16 h	Cornflakes	29
B_2_	B: Ammonium phosphate pH: 5.5	0.008–20	0.01			
B_3_		0.02–30	0.02			
B_6_	Gradient elution, 18 min	0.10–20	0.1			
B_9_		0.03–20	0.03			
B_1_	A: CH_3_(CH_2_)_5_SO_3_Na	18.4–50.4	0.1	Sample + phosphoric acid	Syrup	30
B_2_	B: ACN pH: 2.4–2.5	7.3–21.6	0.2			
B_3_	Elution: Gradient, 60 min	119.8–359	0.2			
B_6_		6.0–17.9	0.1			
B_1_	A: HCOOH	0.07–50	0.04	Sample in phosphoric acid + ultrasonication	Vegetables	31
B_2_	B: ACN C: ACN/MeOH/hexane pH 7.0	0.007–10	0.002			
B_3_		0.03–50	0.01			
B_6_	Elution: Ternary gradient	0.03–50	0.01			
B_9_		0.02–20	0.005			
B_1_	A: 0.1% H_3_PO_4_	0.25–20	0.08	Sample in 0.1 M HCl + ultrasonication	Green leafy vegetables	Present method
B_2_	B: MeOH	0.2–20	0.06			
B_3_	Elution: Gradient	0.2–20	0.06			
B_6_	20 min	0.5–20	0.15			
B_9_		0.4–20	0.10			

Application for determination of WSVs in GLVs. HPLC was employed to analyze the concentration of WSVs in GLVs from the Chhattisgarh region, India. The results revealed substantial variation in vitamin concentrations across different GLVs (Table 3). The concentration range of vitamin B_1_ was 0.12–0.85 mg/100 g, with an average concentration of 0.438 mg/100 g. The highest B_1_ content (0.85 mg/100 g) was found in *Chenopodium album* L. (Bathua Bhaji), while the lowest (0.12 mg/100 g) was observed in *Chorchorus olitorius* L. *Allium cepa* L. (Pyaj Bhaji). Vitamin B_2_ ranged from 0.08–0.44 mg/100 g, with highest amount in *Carthamus tinctorius* L. (Kusum Bhaji) and the lowest in *Cassia tora* (L.) *Roxb.* (Charota). Vitamin B_3_ content was ranged from ND to 2.64 mg/100 g, and highest in *Chenopodium album* L. (Bathua Bhaji) of 2.64 mg/100 g. Vitamin B_6_ ranged from 0.08–0.64 mg/100 g, with the highest in *Carthamus tinctorius* L. (Kusum Bhaji). For vitamin B_9_, the highest content was in *Cordia dichotoma* (Bohar Bhaji) (0.08 mg/100 g), and ranging from ND–0.08 mg/100 g, with an average of 0.04 mg/100 g.

**Table 3:** Determination of WSVs (mg/100 g) in GLVs of Chhattisgarh region

Local name (Bhaji)	Botanical	B_1_	B_2_	B_3_	B_6_	B_9_
Amari	*Hibiscus sabdariffa* L.	0.70	0.27	1.76	0.31	0.02
Bohar	*Cordia dichotoma*	0.26	0.10	0.99	0.38	0.08
Chaulai	*Amaranthus viridis* L.	0.18	0.20	0.94	0.49	ND
Chunchunia	*Marsilea vestita*	0.68	0.16	1.15	0.54	0.03
Charota	*Cassia tora* (L.)*Roxb*	0.40	0.08	1.09	0.27	0.01
Chech	*Chorchorus olitorius* L.	0.22	0.14	ND	0.08	ND
Karmata	*Ipomea aquatica*	0.44	0.40	0.23	0.09	ND
Kusum	*Carthamus tinctorius* L.	0.41	0.44	1.95	0.64	0.05
Kanda	*Ipomea batatas* L.*Lam*	0.26	0.15	1.32	0.48	0.01
Lal	*Amaranthus tricolor* L.	0.44	0.18	0.66	0.34	0.06
Lakdi	*Lathyrus sativus* L.	0.63	0.30	1.59	0.51	0.07
Methi	*T. foenumgraecum* L	0.52	0.19	0.96	0.53	0.04
Palak	*Spinacia oleracea* L.	0.20	0.24	1.62	0.53	0.04
Pyaj	*Allium cepa* L.	0.12	0.13	0.32	0.34	0.04
Poi	*Basella rubra* L.	0.68	0.16	1.15	0.07	ND
Patwa	*Hibiscus cannbinus* L.	0.47	0.21	0.76	0.30	0.07
Bathua	*Chenopodium album* L.	0.85	0.34	2.64	0.39	0.06

The leafy vegetables from Chhattisgarh, such as *Chenopodium album* L. (Bathua Bhaji) (B_1_: 0.85 mg/100 g, B_3_: 2.64 mg/100 g) and *Carthamus tinctorius* L. (Kusum Bhaji) (B_2_: 0.44 mg/100 g, B_6_: 0.64 mg/100 g), showed significantly higher concentrations of WSVs compared to globally popular *Spinacia oleracea* L. (Palak Bhaji) (B_1_: 0.20 mg/100 g, B_6_: 0.53 mg/100 g) and *Amaranthus tricolor* L. (Lal Bhaji) (B_2_: 0.18 mg/100 g, B_6_: 0.34 mg/100 g). These findings highlight the superior vitamin content of local varieties, indicating their potential as rich, underutilized sources of essential nutrients for dietary diversification and improved nutritional security.

### Estimated daily intake

The EDI of WSVs in leafy vegetables (Bhaji) in the Chhattisgarh region is crucial for assessing their nutritional value. The EDI of WSVs (Table 4) of the Chhattisgarh region is compared with the recommended dietary intake (RDI) for men and women recommended by the ICMR (Table S4).^19^ This comparison helped to assess if local diets meet essential vitamin requirements for optimal health. For vitamin B_1_, *Chenopodium album* (Bathua Bhaji) and *Marsilea vestita* (Chunchunia Bhaji) exhibited EDI values of 1.3–1.5 μg per kg per day, which translates to approximately 65–75% of the RDI for women (1.8 mg per day) and about 60–70% for men (2.0 mg per day), indicating that these GLVs were substantial sources of B_1_. Vitamin B_2_, though generally lower across samples, was found in higher concentrations in *Carthamus tinctorius* (Kusum Bhaji) and *Amaranthus viridis* (Chaulai Bhaji), contributing 0.6–0.8 μg per kg per day. However, this accounts for less than 30% of the RDI (2.5–2.7 mg per day), suggesting the need for dietary supplementation from other sources. For vitamin B_3_, Bathua again demonstrated the highest EDI value (4.1–4.8 μg per kg per day), contributing around 20–25% of the RDI for men (20 mg per day) and women (18 mg per day). Vitamin B_6_ showed more promising results, with *Carthamus tinctorius* L. (Kusum Bhaji) and *Spinacia oleracea* (Palak Bhaji) yielding EDI values up to 1.1 μg per kg per day, meeting over 40% of daily requirements. For B_9_, although values were lower across most samples, vegetables like *Lathyrus sativus* (Lakdi bhaji) and Patwa Bhaji contributed about 20–30% of the RDI. These results highlight the value of underutilized regional GLVs, whose regular and combined consumption can help combat micronutrient deficiencies and support dietary diversity in public health and nutrition programs.

**Table 4:** Determination of EDI (μg per kg per day) in green leafy vegetables of Chhattisgarh

Local names of GLVs (Bhaji)	Botanical names	B_1_		B_2_		B_3_		B_6_		B_9_	
		Men	Women	Men	Women	Men	Women	Men	Women	Men	Women
Amari	*Hibiscus sabdariffa* L.	1.0	1.2	0.4	0.5	2.7	3.2	0.5	0.6	0.03	0.04
Bohar	*Cordia dichotoma*	0.4	0.5	0.1	0.2	1.5	1.8	0.6	0.7	0.12	0.14
Chaulai	*Amaranthus viridis* L.	0.2	0.3	0.3	0.4	1.4	1.7	0.7	0.9	—	—
Chunchunia	*Marsilea vestita Hook*	1.0	1.2	0.2	0.3	1.7	2.0	0.8	1.0	0.04	0.05
Charota	*Cassia tora* (L.)*Roxb*	0.6	0.7	0.1	0.1	1.6	1.9	0.4	0.5	0.02	0.02
Chech	*Chorchorus olitorius* L.	0.3	0.4	0.2	0.3	—	—	0.1	0.1	—	—
Karmata	*Ipomea aquatica Forssk*	0.6	0.8	0.6	0.7	0.3	0.4	0.1	0.2	—	—
Kusum	*Carthamus tinctorius* L.	0.6	0.7	0.6	0.8	3.0	3.5	1.0	1.1	0.08	0.09
Kanda	*Ipomea batatas* L.	0.4	0.4	0.2	0.3	2.0	2.4	0.7	0.8	0.02	0.02
Lal	*Amaranthus tricolor* L.	0.6	0.8	0.2	0.3	1.0	1.2	0.5	0.6	0.09	0.11
Lakdi	*Lathyrus sativus* L.	0.9	1.1	0.5	0.5	2.4	2.8	0.8	0.9	0.10	0.12
Methi	*Trigonella foenumgraecum* L	0.8	0.9	0.3	0.3	1.4	1.7	0.8	1.0	0.06	0.07
Palak	*Spinacia oleracea* L.	0.3	0.4	0.4	0.4	2.5	2.9	0.8	1.0	0.06	0.07
Pyaj	*Allium cepa* L.	0.2	0.22	0.2	0.2	0.5	0.5	0.5	0.6	0.06	0.07
Poi	*Basella rubra* L.	1.0	1.2	0.2	0.3	1.7	2.0	0.1	0.1	—	—
Patwa	*Hibiscus cannbinus* L.	0.7	0.9	0.3	0.4	1.2	1.3	0.5	0.5	0.11	0.11
Bathua	*Chenopodium album* L.	1.3	1.5	0.5	0.6	4.1	4.8	0.6	0.7	0.09	0.09

Comparison of vitamins in GLVs with other leafy vegetables across the World. The WSVs in GLVs of Chhattisgarh (Table 3) demonstrated significantly higher concentrations to GLVs^33–39^ from other parts of the world (Table 5). For B_1_, *Chenopodium album* (Bathua, 0.85 mg/100 g) far exceeded the turnip tops (0.19 mg/100 g),^33^ swiss chard (0.04 mg/100 g), spinach (0.12 mg/100 g), and lettuce (0.11 mg/100 g).^36^ In B_2_, *Carthamus tinctorius* (Kusum, 0.44 mg/100 g) and *Ipomea aquatica* (Karmata, 0.40 mg/100 g) outperformed red mustard (0.028 mg/100 g) and garden cress (0.122 mg/100 g).^36^ B_3_ levels in *Chenopodium album* L. (Bathua, 2.64 mg/100 g) and *Carthamus tinctorius* (Kusum, 1.95 mg/100 g) were substantially higher than pea leaves (0.104 mg/100 g).^36^ For B_6_, *Carthamus tinctorius* L. (Kusum, 0.64 mg/100 g) and *Marsilea vestita* (Chunchunia, 0.54 mg/100 g) exceeded the red amaranth (0.152 mg/100 g)^35^ and thankuni leaves (0.13 mg/100 g).^39^ Although B_9_ in Chhattisgarh GLVs was modest (*e.g.*, 0.08 mg/100 g in *Cordia dichotoma*), it still surpassed values in spinach (0.001 mg/100 g) and lambs lettuce (0.006 mg/100 g).^36^ These results highlighted the exceptional micronutrient richness of underutilized Chhattisgarh vegetables, advocating their integration into dietary programs to alleviate global micronutrient deficiencies.

**Table 5:** WSVs (mg/100 g) in GLVs from different region across the World

Common name	Botanical name	Country	B_1_	B_2_	B_3_	B_6_	B_9_	Ref.
Turnip tops	*Brassica rapa*	Spain	0.19	0.200	—	—	—	33
Swiss chard	*Beta vulgaris* L.		0.04	0.040	—	—	—	
Spinach	*Spinacia oleracea* L.		0.12	0.090	—	—	—	
Green lettuce	*Lactuca sativa*		0.11	0.05	—	—	—	
Chacrona	*Psychotria* sp	Nigeria	—	0.04	—	—	—	34
Iyana ipaja	*C. aconitifolius*		—	0.06	—	—	—	
Fluted pumpkin	*T. occidentals*		—	0.08	—	—	—	
Misbredie	*Amaranthus tricolor*	South Africa	—	0.03	—	—	—	35
Pumpkin leaves	*Cucurbita maxima*		—	0.12	—	—	—	
Cowpea leaves	*Vigna unguiculata*		—	0.05	—	—	—	
Cat's whiskers	*Cleome gynandra*		—	0.08	—	—	—	
Wild jute	*Corchorus tridens*		—	0.07	—	—	—	
Pea leaves	*Pisum sativum*	Spain	0.189	0.128	0.104	0.48	—	36
Red mustard	*Brassica juncea*		0.018	0.028	0.067	0.004	—	
Garden cress	*Lepidium sativum*		0.102	0.122	0.159	0.017	—	
Swiss chard	*Beta vulgaris* L.		0.011	0.111	0.139	0.046	—	
Green lettuce	*Lactuca sativa*		0.080	0.030	0.080	0.003	—	
Spinach	*Spinacia oleracea* L.		0.243	0.223	0.178	0.013	0.001	
Lamb's lettuce	*Valeriana locusta*		0.130	0.111	0.193	0.008	0.006	
Bottle gourd leaves	*Lagenaria vulgaris*	Bangladesh	—	0.321	—	0.755	—	37
Green amarnath leaves	*Amarnathus virdis*		—	0.523	0.051	0.07	—	
Red amarnath leaves	*Amarathus gangeticuss*		—	0.442	0.016	0.152	—	
Spinach	*Basella alba*		—	0.397	0.023	—	—	
Bitter gourd leaves	*Momordica charantia*		—	0.137	0.512	—	—	
Bitter leaves	*Vernonia amygdalina*	Nigeria	0.28	0.72	—	—	—	38
Afang	*Gnetum africanum*		0.58	0.72	—	—	—	
Gbure	*Talinium triangulare*		0.55	0.76	—	—	—	
Ugu	*Telfairia occidentalis*		0.75	1.17	—	—	—	
Thankuni leaves	*Centella asiatica*	Bangladesh	0.19	0.250	0.13	0.13	—	39
Mint leaves	*Mentha arvensis*		—	0.060	0.04	—	—	
Coriander leaves	*Coriandrum sativum*		—	—	0.59	—	—	

## Conclusions

This study introduces a new, efficient, and rapid RP-HPLC-DAD method for the simultaneous determination of five essential vitamins (B_1_, B_2_, B_3_, B_6_, and B_9_) in GLVs sourced from Chhattisgarh, India. The methods major innovation lies in its simplified extraction protocol using acid-assisted ultrasonication prior to HPLC analysis, eliminating the need for enzymatic digestion or thermal processing, which often lead to vitamin degradation. The use of 0.1 M HCl and optimized chromatographic conditions provided excellent separation and reproducibility, as evidenced by high recovery rates, and good linearity. By analysing 17 traditional leafy vegetables, the study not only establishes their vitamin content but also evaluates their contribution to dietary requirements through EDI estimation.

The findings show considerable variation in vitamin content among GLVs, with *Chenopodium album* L. (Bathua Bhaji) and *Carthamus tinctorius* L. (Kusum Bhaji) emerging as superior sources of B-complex vitamins. Compared to globally known GLVs like spinach and red amaranth, many indigenous vegetables from Chhattisgarh exhibit higher concentrations of certain vitamins, particularly B_2_ and B_6_. This reinforced the value of integrating these nutrient-dense, underexploited greens into mainstream diets. The study holds significant implications for nutritional security, public health, and food-based strategies in India.

## Conflicts of interest

There are no conflicts to declare.

## Supplementary Material

RA-015-D5RA04911C-s001

## Data Availability

Data is provided as a supplementary material (SI). Supplementary information is available. See DOI: https://doi.org/10.1039/d5ra04911c.

## References

[cit1] Eitenmiller R. R. , Landen Jr W. and Ye L., Vitamin Analysis for the Health and Food Sciences, CRC press, 2016

[cit2] Blake C. J. (2007). Anal. Bioanal. Chem..

[cit3] Gupta U. C., Gupta S. C. (2015). Curr. Nutr. Food Sci..

[cit4] Beitz R., Mensink G. B. M., Fischer B., Thamm M. (2002). Eur. J. Clin. Nutr..

[cit5] Patle T. K., Shrivas K., Patle A., Patel S., Harmukh N., Kumar A. (2022). Microchem. J..

[cit6] Ulusoy H. İ., Acıdereli H., Ulusoy S., Erdoğan S. (2017). Food Anal. Methods.

[cit7] Cellar N. A., McClure S. C., Salvati L. M., Reddy T. M. (2016). Anal. Chim. Acta.

[cit8] Nurit E., Lyan B., Piquet A., Branlard G., Pujos-Guillot E. (2015). Anal. Bioanal. Chem..

[cit9] Selvakumar L. S., Ragavan K. V., Abhijith K. S., Thakur M. S. (2013). Anal. Methods.

[cit10] Pereira D. F., Santana E. R., Spinelli A. (2022). Microchem. J..

[cit11] Wang H., Zhang X., Wang S., Xiao S., Ma H., Wang X. (2020). Microchem. J..

[cit12] Arella F., Lahély S., Bourguignon J. B., Hasselmann C. (1996). Food Chem..

[cit13] Oh Y., Kim J., Cao D., Kim C., Boo K. (2021). Int. Food Res. J..

[cit14] Płonka J., Toczek A., Tomczyk V. (2012). Food Anal. Methods.

[cit15] Khalkho B. R., Kurrey R., Deb M. K., Shrivas K., Thakur S. S., Pervez S., Jain V. K. (2020). Heliyon.

[cit16] Leporati A., Catellani D., Suman M., Andreoli R., Manini P., Niessen W. M. A. (2005). Anal. Chim. Acta.

[cit17] Datta S., Sinha B. K., Bhattacharjee S., Seal T. (2019). Heliyon.

[cit18] Tikeshwari K., Shrivas S., Patel M., Kant T., Thakur S. S., Pervez S., Deb M. K., Ghosh K. K. (2023). ACS Food Sci. Technol..

[cit19] Group I.-N. E. , ICMR-National Institute of Nutrition, Hyderabad, 2020

[cit20] Buszewski B., Noga S. (2012). Anal. Bioanal. Chem..

[cit21] Seal T., Chaudhuri K. (2017). Int. J. Curr. Microbiol. Appl. Sci..

[cit22] Wang H., Helliwell K., You X. (2000). Food Chem..

[cit23] Riyadi P., Susanto E., Anggo A., Arifin M., Rizki L. (2023). Food Res..

[cit24] Hälvin K., Paalme T., Nisamedtinov I. (2013). Anal. Bioanal. Chem..

[cit25] van den Berg H., van Schaik F., Finglas P. M., de Froidmont-Görtz I. (1996). Food Chem..

[cit26] Ciulu M., Solinas S., Floris I., Panzanelli A., Pilo M. I., Piu P. C., Spano N., Sanna G. (2011). Talanta.

[cit27] Gliszczyńska-Świgło A., Rybicka I. (2015). Food Anal. Methods.

[cit28] Viñas P., López-Erroz C., Balsalobre N., Hernández-Córdoba M. (2003). J. Chromatogr. A.

[cit29] Langer S., Lodge J. K. (2014). J. Chromatogr. B.

[cit30] Vidović S., Stojanović B., Veljković J., Pražić-Arsić L., Roglić G., Manojlović D. (2008). J. Chromatogr. A.

[cit31] Melfi M. T., Nardiello D., Cicco N., Candido V., Centonze D. (2018). J. Food Compos. Anal..

[cit32] Sami R., Li Y., Qi B., Wang S., Zhang Q., Han F., Ma Y., Jing J., Jiang L. (2014). J. Chem..

[cit33] del Carmen Mondragón-Portocarrero A., Vázquez-Odériz L., Romero-Rodríguez M. (2011). J. Food Sci..

[cit34] Otitoju G., Ene-Obong H., Otitoju O. (2014). J. Food Process. Technol..

[cit35] Schönfeldt H. C., Pretorius B. (2011). J. Food Compos. Anal..

[cit36] Santos J., Mendiola J. A., Oliveira M. B. P. P., Ibáñez E., Herrero M. (2012). J. Chromatogr. A.

[cit37] Hasan M. N., Akhtaruzzaman M., Sultan M. Z. (2013). J. Anal. Sci. Methods Instrum..

[cit38] Adebayo O. A. (2019). Act. Sci. Nutr. Health.

[cit39] Mozumder N. R., Akhter M. J., Khatun A. A., Rokibuzzaman M., Akhtaruzzaman M. (2019). Orient. J. Chem..

