# Supplementary material for: RP-HPLC for simultaneous determination of water-soluble vitamins in leafy vegetables using ultrasonicated acid hydrolysis
Source: RSC Adv. 2025 Oct 14;15(45):38270–80. doi: 10.1039/d5ra04911c (PMC12519113; doi:10.1039/d5ra04911c)
Supplement: RA-015-D5RA04911C-s001 [file RA-015-D5RA04911C-s001.pdf]

Supplementary Information (SI) for RSC Advances.
This journal is © The Royal Society of Chemistry 2025

Supplementary Information

RP-HPLC for Simultaneous Determination of Five Water-Soluble Vitamins in Green Leafy Vegetables using Ultrasonicated Acid Hydrolysis

Tikeshwari,[1,2] Kamlesh Shrivas[1*], Khushali Tandey[1], Ankita Tejwani[1], Arun Kumar Patel[1,3], and Anuradha Sharma[4]

[1]School of Studies in Chemistry, Pt. Ravishankar Shukla University, Raipur-492010, CG, India

[2]Chandrapal Dadsena Govt. College Pithora-493551, Mahasamund, CG, India

[3]School of Studies in Electronics, Pt. Ravishankar Shukla University, Raipur- 492010, CG, India

[4]Department of Zoology, Govt. Nagarjuna P.G. College of Science, Raipur-492010, CG, India

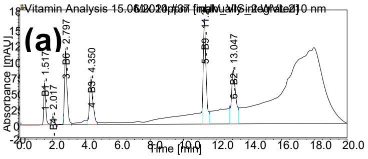

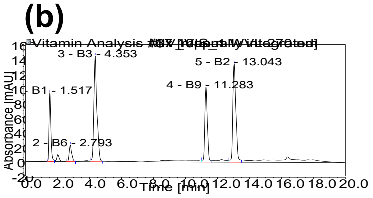

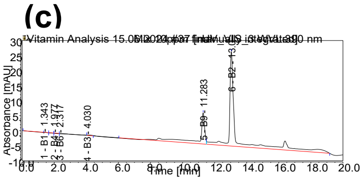

Fig. S1. Chromatographic peak of water-soluble vitamins in HPLC-DAD at different wavelengths (a) 210 nm, (b) 270 nm and 390 nm

(a)

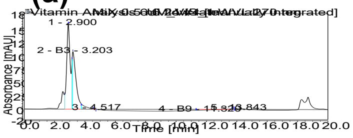

(b)

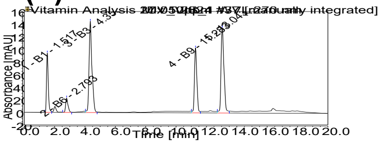

(c)

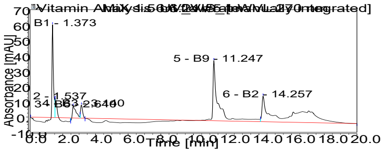

Fig. S2. Chromatographic peak of water-soluble vitamins in HPLC-DAD at different flow rate (a) 0.5 mL/min, (b) 1.0 mL/min, and (c) 1.5 mL/min at 270 nm wavelength

(a)

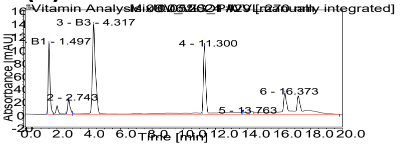

(b)

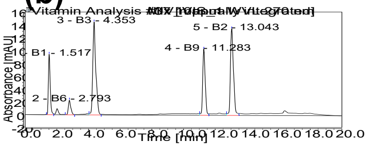

(c)

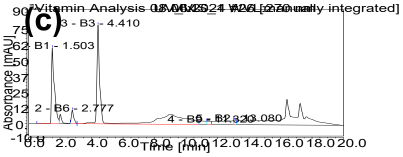

Fig. S3. Chromatographic peak of water-soluble vitamins in HPLC-DAD at different concentrations of OPA (a) 0.05% (b) 0.1%, and (c) 0.2%

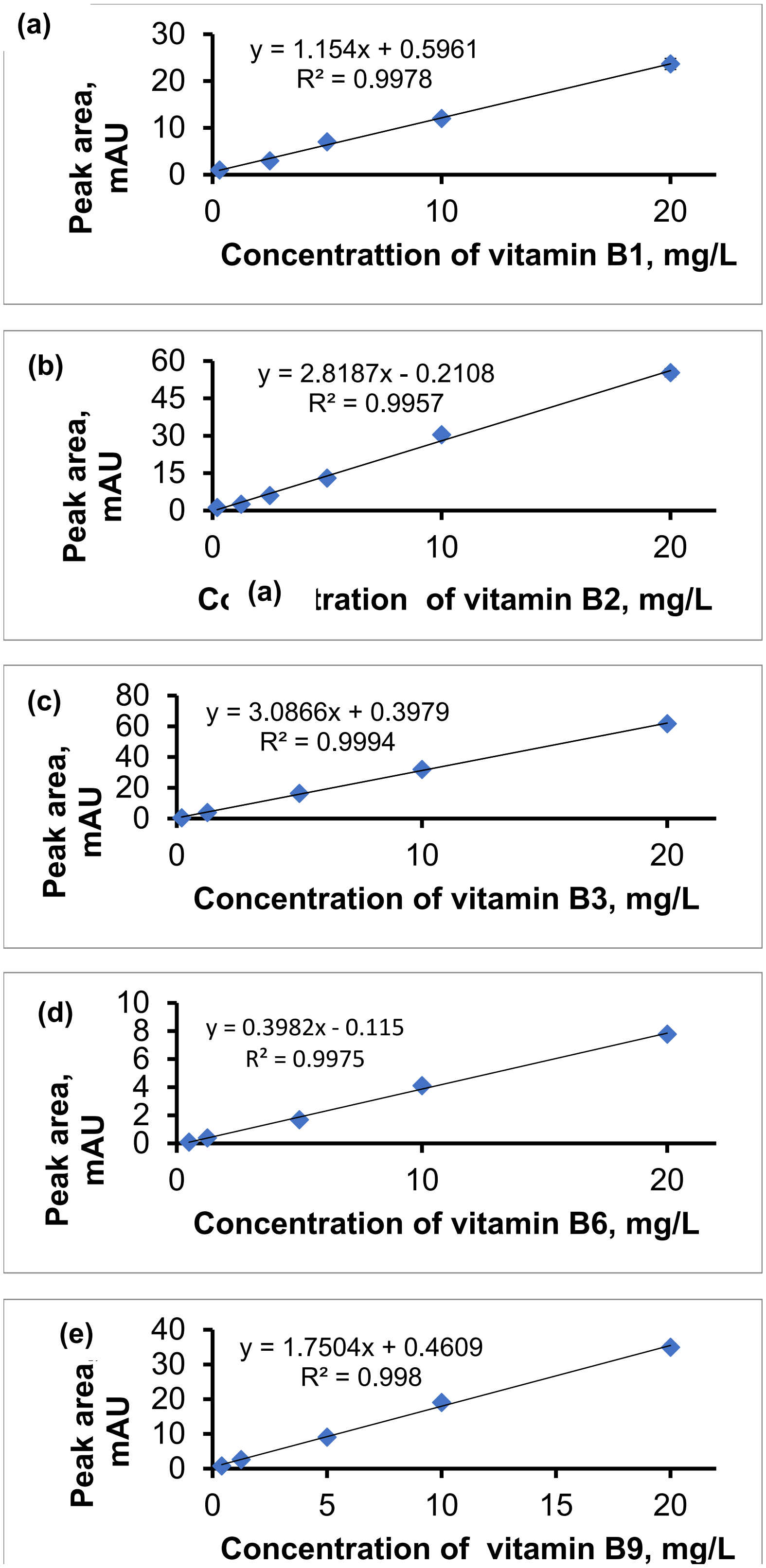

Fig. S4. Standard calibration of vitamins (a) B_1, (b) B_2, (c) B_3, (d) B_6 and (e) B_9 using RP-HPLC, at flow rate of 1.0 mL/min and detection at 270 nm.

**Table S1.** GLVs of Chhattisgarh showing local name, botanical name, family, ecological occurrence, season of availability, medicinal properties						
S. No.	Local Name (Bhaji)	Botanical name	Family	Ecological Occurrence	Season of Availabilit y	Benefits
1	Amari	*Hibiscus sabdariffa L.*	Malvaceae	Cultivated	Summer, Rainy	Rich source of iron, vitamin C, good for human health (Prevents heavy bleeding during menstruation), maintaining strong and healthy bone, boosting the immune system, Control blood sugar levels, prevents constipation, stomach soother
2	Bohar	*Cordia dichotoma*	Boraginaceae	Cultivated	Summer	It is rich in iron, fibre, and essential nutrients, which help boost immunity. traditional beliefs suggest that it aids digestion and helps keep the body cool.
3	Chaulai	*Amaranthus viridis L.*	Amaranthaceae	Cultivated	Year around	Diuretic, analgesic, antipyretic, antiulcer, antichlorsterolemic, asthma and veneral disease
4	Chunchunia	*Marsilea vestita*	Marsileaceae	Weed	Wet moist soil Year around	Anti-inflammatory, diuretic, depurative, febrifuge, and refrigerant, it is also used to treat snake bite
5	Charota	*Cassia tora (L.) Roxb.*	Fabaceae	Weed in road sides Fields	Rainy	Ring worm, leprosy, itching, snake bites, cardiac disorders
6	Chech	*Chorchorus trilocularis L.*	Tiliaceae	Cultivated	Whole Year	The leaves are used as a plaster to reduce swellings
7	Karmota	*Ipomea aquatica*	Convolvulaceae	Ponds and marshy lands	Summer	a amazing leaf vegetables it is able to reduce blood pressure, gives immunity to cancer, improve vision and treat skin disease

8	Kusum	*Carthamus tinctorius L.*	Asteraceae	Cultivated in small scale	Winter	Reduce the risk of heart disease, thin the blood to prevent clots, widen blood vessels, stimulate the heart
9	Kanda	*Ipomea batatas L. Lam.*	Convolvulaceae	Cultivated areas in hedges	Winter	Rich in calcium and Phosphorus, Treat abdominal disease, antidiabetic, antioxidant
10	Lal Bhaji	*Amaranthus tricolor L.*	Amaranthaceae	Cultivated	Winter	Anti-inflammatory, Intestinal cramps, externally to treat wounds, hepatitis, bronchitis, asthma, lung troubles
11	Lakhdi Bhaji	*Lathyrus sativus L.*	Fabaceae	Cultivated	Winter	Useful in treating paralysis and affections of the spinal cord, deformities like knock knees
12	Methi	*T. foenumgraecum L*	Fabaceae	Cultivated	Winter	The appetite, relieves fever, Antitumor, laxative, carminative, anticarcinogrnic, antidiabetic
13	Palak	*Spinacia oleracea L.*	Amaranthaceae	Cultivated	Winter	Anamia, night blindness, used to treat stomach and intestinal complaints and fatigue, blood builder and an appetite stimulant
14	Pyaj	*Allium cepa L*	Amaryllidaceae	Commercial crop	Rabi, kharif	Sunstroke, vomiting, prevention of cancer, antibacterial, antifungal, Sexual debility
15	Poi	*Basella rubra L.*	Basellaceae	Cultivated areas in hedges	Rainy, Summer	Anti-ulcer, antioxidant, cytotoxic, antibacterial, nephroprotective and wound healing properties
a16	Patwa	*Hibiscus sabdariffa L.*	Malvaceae	Cultivated	Rainy	antioxidant, anti-inflammatory, and antimicrobial activities.
17	Bhathuwa	*Chenopodium album L.*	Amaranthaceae	Cultivated in small scale, Weed in cultivated crops	Winter	Anthelminitic, antiphlogistic, antirheumnatic, bug bites, urinary problem, sunstroke, promotes eye health,

						arcdiotonic, diuretic

Table S2. Gradient elution for the chromatographic separation of WSVs in GLVS using A (OPA) and B (MeOH)

TIME (MIN)	Mobile phases	
	A %	B%
0.0	100	0
1.1	98	2
2.4	96	4
5.6	95	5
6.2	90	10
14	60	40
15	40	60
16.4	40	60
18	100	0
20	100	0

Table S3. Determination of recovery % using HPLC-DAD for analysis of WSVs in leafy vegetable samples

WSVs	Added, µg/mL	Found, µg/mL	Recovery, %
B_1	0.5	0.48	96.0
	2	1.88	94.0
B_2	0.5	0.49	98.0
	2	1.93	96.5
B_3	0.5	0.47	94.0
	2	1.83	91.5
B_6	0.5	0.49	98.0
	2	1.87	93.5
B_9	0.5	0.46	92.0
	2	1.86	93.0

**Table S4.** The recombed daily intake (mg/day) of WSVs [ICMR, 19]		
Vitamins	Female	Male
B_1	1.8	2.0
B_2	2.5	2.7
B_3	18	20
B_6	2.5	2.3
B_9	0.300	0.570
